# Kernel Bayesian nonlinear matrix factorization based on variational inference for human–virus protein–protein interaction prediction

**DOI:** 10.1038/s41598-024-56208-w

**Published:** 2024-03-08

**Authors:** Yingjun Ma, Yongbiao Zhao, Yuanyuan Ma

**Affiliations:** 1 School of Mathematics and Statistics, Xiamen University of Technology, Xiamen, China; 2 School of Computer, Central China Normal University, Wuhan, China; 3 School of Computer Engineering, Hubei University of Arts and Science, Xiangyang, China; 4 Hubei Key Laboratory of Power System Design and Test for Electrical Vehicle, Hubei University of Arts and Science, Xiangyang, China

**Keywords:** Human proteins, Viral proteins, Bayesian matrix factorization, Automatic rank determination, Variational inference, Computational biology and bioinformatics, Machine learning, Network topology, Probabilistic data networks

## Abstract

Identification of potential human–virus protein–protein interactions (PPIs) contributes to the understanding of the mechanisms of viral infection and to the development of antiviral drugs. Existing computational models often have more hyperparameters that need to be adjusted manually, which limits their computational efficiency and generalization ability. Based on this, this study proposes a kernel Bayesian logistic matrix decomposition model with automatic rank determination, VKBNMF, for the prediction of human–virus PPIs. VKBNMF introduces auxiliary information into the logistic matrix decomposition and sets the prior probabilities of the latent variables to build a Bayesian framework for automatic parameter search. In addition, we construct the variational inference framework of VKBNMF to ensure the solution efficiency. The experimental results show that for the scenarios of paired PPIs, VKBNMF achieves an average AUPR of 0.9101, 0.9316, 0.8727, and 0.9517 on the four benchmark datasets, respectively, and for the scenarios of new human (viral) proteins, VKBNMF still achieves a higher hit rate. The case study also further demonstrated that VKBNMF can be used as an effective tool for the prediction of human–virus PPIs.

## Introduction

Viruses are widely distributed in nature and can parasitize in various living organisms, which leads to highly contagious viral diseases, and their prevalence and outbreaks will pose a major threat to human life and health. In the past ten years, the number of cases of dengue fever in the world has continued to increase. The disease is mainly transmitted by Aedes mosquitoes, and about 390 million people are infected worldwide every year^1^. Since 1976, there have been more than 40 outbreaks of Ebola virus disease, with a fatality rate of between 25 and 90%. The deadliest Ebola outbreak, in West Africa in 2014, produced 28,610 cases and killed 11,308 people, drawing widespread international attention^2,3^. The outbreak of coronavirus disease in 2019 spread rapidly around the world^4^. According to the statistics of the World Health Organization, as of May 10, 2023, more than 700 million people had been diagnosed with the infection, resulting in nearly 7 million deaths, and having an unimaginable impact on the life, health and economic security of all mankind. Studies have shown that virus-host PPIs are the main way for viruses to exercise their functions. This interaction is very durable, starting from the binding of viral coat proteins to host membrane receptors and continuing until viral proteins control the host transcription system^5,6^. Therefore, the exploration of human–viral PPIs contributes to the understanding of the pathogenesis of viruses and provides the necessary foundation for the development of effective treatment and prevention strategies to combat viral diseases.

At present, high-throughput experimental techniques such as yeast two-hybridization (Y2H) and mass spectrometry (MS) have been widely used in protein function inference and biological process research^7^. However, these methods are mainly used to identify intraspecific PPIs, and there are few studies on interspecific PPIs. In addition, experimental methods are not only time-consuming and laborious, but also difficult to obtain a complete protein interactome^8^. As the number of virus-host PPIs continues to increase, computational models for the prediction of interspecies PPIs have also received increasing attention^9^. Yang et al.^8^ utilized doc2vec to represent protein sequences as rich low-dimensional feature vectors, and used random forests to perform predictions, and the results showed that the prediction performance of this method was better than that of SVM, Adaboost and Multiple Layer Perceptron. Yang et al.^10^ combined evolutionary sequence features with Siamese convolutional neural network architecture and multi-layer perceptron, introduced two transfer learning methods (namely "frozen" type and "fine-tuned" type), and successfully applied them to the prediction of virus–human PPIs by retraining CNN layer. To predict potential human–virus PPIs, Tsukiyama et al.^11^ used word2vec to obtain low-dimensional features from amino acid sequences and developed an LSTM-based prediction model. The above supervised learning methods effectively use the sequence information of proteins, and have achieved some success in the prediction of virus–human PPIs. However, most of these methods require negative sampling to generate training sets, which inevitably leads to false negative samples in the training set. In addition, these models often need to ensure a balanced ratio of positive and negative samples when performing training, and do not make full use of a large number of other unknown interactions, which also limits the predictive ability of the models to a certain extent.

In recent years, more and more network models for predicting interaction relationships have been proposed. Based on multiple similarity kernels for viral (or human) proteins, Nourani et al.^12^ proposed an adaptive multi-kernel preservation embedding (AMKPE) approach to perform predictions. The results show that AMKPE achieves better performance than some supervised learning methods. In the previous study, we proposed a sequence ensemble-based virus–human PPIs prediction method (Seq-BEL)^13^, which integrated sequence feature information and network structure into the ensemble learning model to improve the prediction ability and stability. Recently, for the prediction of human–virus PPIs under various disease types, we proposed a logical tensor decomposition model with sparse subspace learning^14^, which introduced logical functions and feature information into CP decomposition to improve the prediction ability of human–virus-disease triples. In addition, some other binary interaction prediction methods also provide reference for the prediction of virus–human PPIs. Peska et al.^15^ proposed a Bayesian ranking model for predicting drug–target interactions based on Bayesian personalized ranking matrix factorization, which showed good predictive performance on multiple benchmark datasets. Sharma et al.^16^ proposed a bagging based ensemble framework for drug–target interaction prediction, which employ reduction and active learning to deal with class imbalance data, showing excellent performance compared with other five competing methods. Ding et al.^17^ proposed a dual Laplacian regularized least squares (DLapRLS) model for drug–target interaction prediction, which utilized the Hilbert–Schmidt Independence Criterion-based Multiple Kernel Learning (HSIC-MKL) to linearly integrate the corresponding kernels in drug space and target space, respectively, and established a drug–target interactive prediction model by DLapRLS. Yu et al.^18^ proposed an end-to-end graph deep learning approach (LAGCN) that utilized GCN to capture structural information from heterogeneous networks of drugs and diseases, and introduced attentional mechanisms to combine embeddings from different convolutional layers for drug-disease association prediction. Zhao et al.^19^ proposed an improved Graph representation learning method (iGRLDTI), which solves the oversmoothing problem of graph neural networks (GNN) by better capturing the more discriminant features of drugs and targets in the potential feature space. The above model makes full use of the network structure of biological entities and improves the predictive ability of the model. However, most of the above models contain more hyperparameters, and the parameter adjustment before the experiment affects the prediction efficiency and generalization ability of the model to a certain extent.

Therefore, this study proposes a kernel Bayesian nonlinear matrix factorization based on variational inference, VKBNMF, for human–virus PPIs prediction. To reduce the sparsity of the interaction network and improve the accuracy of the similarity network, we extract the kernel neighborhood similarity from the completed virus–human PPIs network, and fused it with the sequence similarity of the viral (or human) protein to obtain a more accurate network structure. Secondly, to improve the learning ability of the model, we introduce the weighted logistic function into kernel Bayesian Matrix Factorization, and adaptively determine the rank of low-dimensional features by combining the sparsity-inducing priors of multiple latent variables. Finally, to solve the problem of integrating latent variables and ensuring the efficiency of the solution, we establish a variational inference framework to implement the model solution. Results on three experimental scenarios in four real data sets demonstrate the effectiveness of VKBNMF in predicting potential human–viral PPIs. Furthermore, the case study further demonstrates that VKBNMF can be used as an effective tool for human–viral PPIs prediction.

## Methods

### Method review

**Figure 1:**
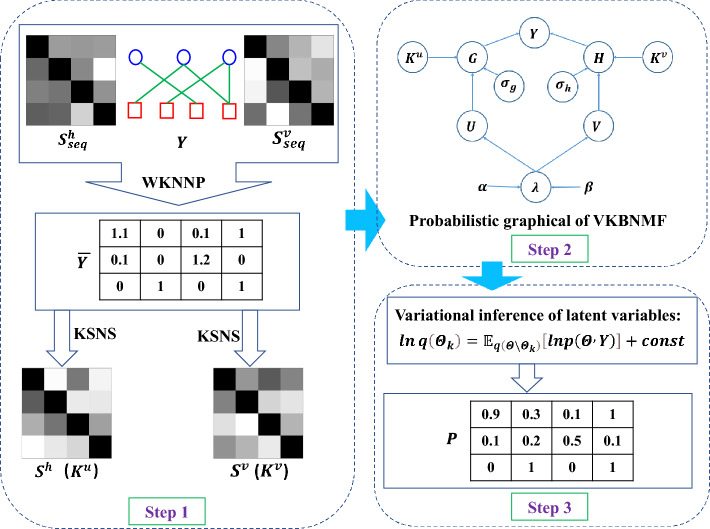
The overall workflow of VKBNMF for predicting of potential human–virus PPIs.

To explore virus–human potential PPIs, we propose a new method named VKBNMF, which mainly consists of three steps (as shown in Fig. 1). Firstly, a variety of similarity networks are constructed based on protein sequences and trained human–virus PPIs networks, and are fused to obtain more accurate similarity of viral (or human) proteins (as shown in step 1 of Fig. 1). Secondly, the Bayesian framework of logical matrix factorization is established, and the auxiliary information of human (or viral) protein and the prior probabilities of latent variables are introduced, and then the probability graph model of VKBNMF is constructed (as shown in step2 in Fig. 1). Finally, variational inference is used to perform the solution of VKBNMF to realize the prediction of potential PPIs of human–virus (as shown in step 3 in Fig. 1).

### Network construction

Let $Y\in {\mathbb{R}}^{M\times N}$ represent the interaction matrix of $M$ human proteins and $N$ viral proteins. When there is an interaction between the *i*th human protein and the *j*th viral protein, then ${Y}_{ij}=1$, otherwise ${Y}_{ij}=0$. ${S}_{seq}^{h}$ (or ${S}_{seq}^{v}$) represents sequence similarity of human (or viral) proteins, respectively. The task at hand is to predict potential interactions in $Y$.

According to previous research, reasonably extracting information from known interaction networks can enhance the accuracy of the network, thereby improving the predictive ability of the model^13,20–22^. However, the existing interactive networks are very sparse, and the information contained is more focused on well-studied samples, and extracting information directly from them will contain more noise. Therefore, drawing on the method of Xiao et al.^23^, based on ${S}_{seq}^{h}$ and ${S}_{seq}^{v}$, we utilize weighted k nearest neighbor profiles (WKNNP) to initially complete the trained $Y$ to obtain $\overline{Y }$. In previous studies, we proposed a network construction method based on kernel neighborhood similarity (KSNS)^24,25^, which can hierarchically integrate neighborhood and non-neighborhood information and mine nonlinear relationships of samples, and has been well applied in some biological relationship prediction problems^20,21,26,27^. KSNS calculates the similarity as follows:

1 $$\begin{aligned} &\underset{W\ge 0}{{{\max}}}\left\{\frac{1}{2}{\Vert \phi \left(X\right)W-\phi \left(X\right)\Vert }_{F}^{2}+\frac{{\mu }_{1}}{2}{\Vert W\odot \left(1-C\right)\Vert }_{F}^{2}+\frac{{\mu }_{2}}{2}{\Vert W\Vert }_{F}^{2}\right\}\\ &\quad s.t. {\sum }_{i}{W}_{ij}=1, i={1,2},\ldots ,n\end{aligned}$$

where $\Phi \left(\cdot \right)$ represents kernal transformation, and Gaussian function is selected in this paper. ${\| \cdot \| }_{F}$ denotes *F*-norm, and ⨀ is an element-by-element multiplication. ${\mu }_{1}$ and ${\mu }_{2}$ represent regularization parameters, according to previous studies^21,27,28^, ${\mu }_{1}=4$ and ${\mu }_{2}=1$. According to (1), when $X=\overline{Y }$, the interaction profile similarity ${S}_{int}^{h}$ of human protein can be obtained; when $X={\overline{Y} }^{T}$, the interaction profile similarity ${S}_{int}^{v}$ of viral proteins can be obtained.

Then, we obtain two similarities of human proteins (${S}_{seq}^{h},{S}_{int}^{h}$) and two similarities of viral proteins (${S}_{seq}^{v}$, ${S}_{int}^{v}$), which both measure the relationship of human (or viral) proteins from different aspects. To obtain a more accurate network structure, clusDCA^29^ is used to fuse ${S}_{seq}^{h}$ and ${S}_{int}^{h}$ to obtain the final human protein similarity ${S}^{h}$, and ${S}_{seq}^{v}$ and ${S}_{int}^{v}$ to obtain the final viral protein similarity ${S}^{v}$.

### VKBNMF

Liu et al.^30^ introduced neighborhood similarity into logical matrix factorization, and obtained a neighborhood regularized logical matrix factorization model (NRLMF), which and its variants are well applied to the interaction relationship prediction of various biological entities^28,30,31^. However, NRLMF needs to undergo tedious hyperparameter tuning before performing prediction tasks, which not only affects computational efficiency, but may also lead to overfitting. This paper establishes a Bayesian framework based on LMF, takes hyperparameters as latent variables, and introduces prior probability, so that the model can adaptively search for the optimal solution, avoid tedious hyperparameter debugging, and improve prediction performance and generalization ability.

**Figure 2:**
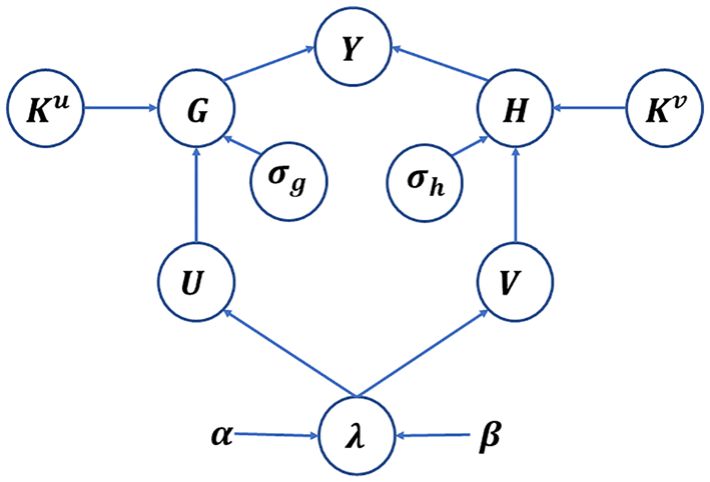
Directed graph representation of VKBNMF model.

Let $G\in {\mathbb{R}}^{M\times R}$ and $H\in {\mathbb{R}}^{N\times R}$ represent the factor matrices of human proteins and viral proteins respectively, then the interaction relationship between the $m$ th human protein and the *n*th viral protein satisfies the Bernoulli distribution, and the density function can be expressed as:

2 $$P({Y}_{m,n}|{G}_{m.},{H}_{n.})={\sigma \left({G}_{m.}{{H}_{n.}}^{T}\right)}^{{y}_{m,n}}{\left(1-\sigma \left({G}_{m.}{{H}_{n.}}^{T}\right)\right)}^{1-{Y}_{m,n}}$$

where, $\sigma \left(\cdot \right)$ represents the sigmoid function, ${G}_{m.}$ and ${H}_{n.}$ represent the $m$ th row of $G$ and the $n$ th row of $H$, respectively. NRLMF considers that known interactions are more important and need to be assigned higher weights. Meanwhile, assuming that all training samples are independent, the weighted conditional probability density of $Y$ can be expressed as:

3 $$P(Y|G,H)=\prod_{m=1}^{M}\prod_{n=1}^{N}{\sigma \left({G}_{m.}{{H}_{n.}}^{T}\right)}^{c{Y}_{m,n}}{\left(1-\sigma \left({G}_{m.}{{H}_{n.}}^{T}\right)\right)}^{1-{Y}_{m,n}}$$

where, $c\ge 1$ represents the importance level. Figure 2 demonstrates the probabilistic graphical model of VKBNMF with latent variables and corresponding priors.

From Fig. 2, the probability of occurrence of $Y$ is calculated from the factor matrix $G$ and $H$ by (3). The probability distributions of factor matrices $G$ and $H$ are obtained from $U\in {\mathbb{R}}^{M\times R}$ and $V\in {\mathbb{R}}^{N\times R}$ by integrating two types of auxiliary information ${K}^{u}$ (e.g. ${S}^{h}$) and ${K}^{v}$ (e.g. ${S}^{v}$). ${\sigma }_{g}$, ${\sigma }_{h}$ and ${\varvec{\lambda}}$ are precision parameters, while $\alpha$ and $\beta$ are hyperparameters. In this section, we specify priors on all latent variables and parameters.

In general, the effective dimension $R$ of the latent space (e.g. the effective column dimensions of $U$ and $V$) is a tuning parameter whose selection is quite challenging and computationally expensive. In order to both infer the value of *R* and avoid overfitting, we introduce automatic rank determination into the prior distributions of *U* and *V*^32^. Specifically, it is assumed that each column of *U* and *V* is independent, and its *r*th column satisfies the vector with a mean value of 0, and the precision matrix is the Gaussian prior of ${\lambda }_{r}{I}_{M}$ and ${\lambda }_{r}{I}_{N}$, respectively, as follows:

4 $$P\left(U|{\varvec{\lambda}}\right)=\prod_{r=1}^{R}\mathcal{N}\left({U}_{\cdot r}|0,{{\lambda }_{r}}^{-1}{I}_{M}\right)$$

5 $$P\left(V|{\varvec{\lambda}}\right)=\prod_{r=1}^{R}\mathcal{N}\left({V}_{\cdot r}|0,{{\lambda }_{r}}^{-1}{I}_{N}\right)$$

where ${I}_{M}\in {\mathbb{R}}^{M\times M}$ and ${I}_{N}\in {\mathbb{R}}^{N\times N}$ represent the identity matrix, ${U}_{\cdot r}$ and ${V}_{\cdot r}$ represent the $r$ th column of $U$ and $V$, respectively. $\left[{\lambda }_{1},{\lambda }_{2},\ldots ,{\lambda }_{R}\right]$ constitutes the precision vector ${\varvec{\lambda}}{\in {\mathbb{R}}}^{1\times M}$. ${\lambda }_{r}$ controls the r column of and $V$. When ${\lambda }_{r}$ is large, Ur and Vr both approach 0, indicating that they make little contribution to Y and can be removed from U and V. This process can realize the automatic determination of $R$. For the precision vector ${\varvec{\lambda}}$, the conjugate Gamma hyperprior is defined as follows:

6 $$P\left({\varvec{\lambda}}|\alpha ,\beta \right)=\prod_{r=1}^{R}Gamma\left({\lambda }_{r}|\alpha ,\beta \right)$$

where, $Gamma\left(x|\alpha ,\beta \right)=\frac{{\beta }^{\alpha }}{\Gamma \left(\alpha \right)}{x}^{\alpha -1}{e}^{-\beta x}$ is the Gamma distribution, and $\left\{\alpha ,\beta \right\}$ are the two parameters of the Gamma distribution. In this study, no information prior is selected^33^, that is, $\alpha =1$, $\beta =1$. In order to effectively integrate the auxiliary information, let the elements in the factor matrix $G$ be independent, and the $(m,r)th$ element ${G}_{m,r}$ satisfies the Gaussian distribution with the expectation of ${K}_{m\cdot }^{u}{U}_{\cdot r}$ and precision ${\sigma }_{g}$, as follows:

7 $$P\left(G|U,{K}^{u},{\sigma }_{g}\right)=\prod_{m=1}^{M}\prod_{r=1}^{R}\mathcal{N}\left({G}_{m,r}|{K}_{m\cdot }^{u}{U}_{\cdot r},{{\sigma }_{g}}^{-1}\right)$$

Similarly, according to ${K}^{v}$ and $V$, the prior probability of $H$ is as follows:

8 $$P\left(H|V,{K}^{v},{\sigma }_{h}\right)=\prod_{n=1}^{N}\prod_{r=1}^{R}\mathcal{N}\left({H}_{n,r}|{K}_{n\cdot }^{v}{V}_{\cdot r},{{\sigma }_{h}}^{-1}\right)$$

where, ${\sigma }_{h}$ is the precision parameter. Here, ${\sigma }_{g}$ and ${\sigma }_{h}$ satisfy the Jeffreys prior

9 $$P\left({\sigma }_{g}\right)\propto {{\sigma }_{g}}^{-1}$$

10 $$P\left({\sigma }_{h}\right)\propto {{\sigma }_{h}}^{-1}$$

According to the probability graph model described in Fig. 1, combined with the likelihood function in (3), the priors of $U$ and $V$ in (4) and (5), the priors of precision vector ${\varvec{\lambda}}$ in (6), the priors of factor matrix $G$ and $H$ in (7) and (8), and the priors of precision ${\sigma }_{g}$ and ${\sigma }_{h}$ in (9) and (10), the joint distribution of VKBNMF is given by:

11 $$P\left(Y,G,H,U,V,{\varvec{\lambda}},{\sigma }_{g},{\sigma }_{h}\right)=P\left(Y|G,H\right)P\left(G|U,{K}^{u},{\sigma }_{g}\right)P\left(H|V,{K}^{v},{\sigma }_{h}\right)P\left(U|{\varvec{\lambda}}\right)P\left(V|{\varvec{\lambda}}\right)P\left({\varvec{\lambda}}|\alpha ,\beta \right)P\left({\sigma }_{g}\right)P\left({\sigma }_{h}\right)$$

Let $\Theta =\left\{G,H,U,V,{\varvec{\lambda}},{\sigma }_{g},{\sigma }_{h}\right\}$ represent the set of all potential variables, and our goal is to compute the complete posterior distribution of all potential variables given $Y$

12 $$P\left(\Theta |Y\right)=\frac{P\left(\Theta ,Y\right)}{\int P\left(\Theta ,Y\right)d\Theta }$$

### Model Inference of VKBNMF

The accurate solution of (12) requires the integration of all potential variables, which is computationally intractable. Therefore, this study employs variational inference to obtain the approximate posterior distribution $q\left(\Theta \right)$ for $P\left(\Theta |Y\right)$. The principle of variational inference is to define a set of parameter distributions on latent variables and update the parameters to minimize the Kullback–Leibler (KL) distance between $P\left(\Theta |Y\right)$ and $q\left(\Theta \right)$^34^

13 $$\underset{q\left(\Theta \right)}{{min}}KL\left(q\left(\Theta \right)|P\left(\Theta |Y\right)\right)=\underset{q\left(\Theta \right)}{min}\left\{\int q\left(\Theta \right){ln}\left[\frac{q\left(\Theta \right)}{P\left(\Theta |Y\right)}\right]d\Theta \right\}=lnP\left(Y\right)-\underset{q\left(\Theta \right)}{min}\left\{\int q\left(\Theta \right){ln}\left[\frac{P\left(\Theta ,Y\right)}{q\left(\Theta \right)}\right]d\Theta \right\}$$

where $lnP\left(Y\right)$ represents model evidence and its lower bound is defined as $\mathcal{L}\left(q\right)=\int q\left(\Theta \right){ln}\left\{\frac{P\left(\Theta ,Y\right)}{q\left(\Theta \right)}\right\}d\Theta$. According to the mean field approximation, $q\left(\Theta \right)$ can be decomposed into

14 $$q\left(\Theta \right)=\prod_{k}q\left({\Theta }_{k}\right)=q(G)q(H)q(U)q(V)q({\varvec{\lambda}})q({\sigma }_{g})q\left({\sigma }_{h}\right)$$

When the other variables are fixed, the optimal posterior estimate of $q\left({\Theta }_{k}\right)$ is defined as follows:

15 $${ln}q\left({\Theta }_{k}\right)={\mathbb{E}}_{q\left(\Theta \backslash {\Theta }_{k}\right)}\left[lnp(\Theta ,Y)\right]+const$$

where, ${\mathbb{E}}\left[\cdot \right]$ represents expectation, and $const$ represents a constant that does not depend on the current variable. $\Theta \backslash {\Theta }_{k}$ represents the $\Theta$ set after deleting ${\Theta }_{k}$. All variables are updated sequentially while keeping other variables constant.

1) *Estimate the latent variable* ${\varvec{\lambda}}$: Combining the respective priors of $U$, $V$ and $\lambda$ in (4), (5) and (6), the posterior approximation $Lnq\left({\lambda }_{r}\right)$ is derived from (15) as

16 $$Lnq\left({\lambda }_{r}\right)={\mathbb{E}}_{q\left(\Theta \backslash {\lambda }_{r}\right)}\left[Ln\left\{P\left(U|\lambda \right)P\left(V|\lambda \right)P\left(\lambda |\alpha ,\beta \right)\right\}\right]+const={\mathbb{E}}\left[\left(\frac{M+N+2{\alpha }}{2}-1\right)Ln\left({\lambda }_{r}\right)-\left(\frac{{{U}_{\cdot r}}^{T}{U}_{\cdot r}+{{V}_{\cdot r}}^{T}{V}_{\cdot r}}{2}+\upbeta \right){\lambda }_{r}\right]+const$$

From (16), it is found that the posterior density of the ${\lambda }_{r}$ obey the Gamma distribution

17 $$q\left({\lambda }_{r}\right)=Gamma\left({\lambda }_{r}|{\widetilde{\alpha }}_{r},{\widetilde{\beta }}_{r}\right)$$

where ${\widetilde{\alpha }}_{r}$ and ${\widetilde{\beta }}_{r}$ represent the posterior parameters as follows:

$${\widetilde{\alpha }}_{r}=\frac{M+N+2{\alpha }}{2}$$

18 $${\widetilde{\beta }}_{r}=\frac{{\mathbb{E}}\left({{U}_{\cdot r}}^{T}{U}_{\cdot r}\right)+{\mathbb{E}}\left({{V}_{\cdot r}}^{T}{V}_{\cdot r}\right)}{2}+\upbeta $$

The required expectations here are found as

19 $$\begin{aligned}& {\mathbb{E}}\left({{U}_{\cdot r}}^{T}{U}_{\cdot r}\right)={{\widetilde{U}}_{\cdot r}}^{T}{\widetilde{U}}_{\cdot r}+tr\left(\Sigma \left({U}_{\cdot r}\right)\right)\\ &{\mathbb{E}}\left({{V}_{\cdot r}}^{T}{V}_{\cdot r}\right)={{\widetilde{V}}_{\cdot r}}^{T}{\widetilde{V}}_{\cdot r}+tr\left(\Sigma \left({V}_{\cdot r}\right)\right)\end{aligned}$$

where ${\widetilde{U}}_{\cdot r}$ and ${\widetilde{V}}_{\cdot r}$ represent the posterior expectation of ${U}_{\cdot r}$ and ${V}_{\cdot r}$, respectively. $\Sigma \left({U}_{\cdot r}\right)$ and $\Sigma \left({V}_{\cdot r}\right)$ represent the posterior covariance matrix of ${U}_{\cdot r}$ and ${V}_{\cdot r}$, respectively. $tr\left(\cdot \right)$ represents the trace of a matrix.

2) *Estimate latent variables* $U$ *and* $V$: Substituting the priors of the latent variables $U$ and $G$ into (15), the posterior approximation of $Lnq\left({U}_{\cdot r}\right)$ is obtained as follows (see section 1 of Appendix for details):

20 $$Lnq\left({U}_{\cdot r}\right)={\mathbb{E}}_{q\left(\Theta \backslash {U}_{\cdot r}\right)}\left[Ln\left\{P\left(G|U,{K}^{u},{\sigma }_{g}\right)P\left(U|\lambda \right)\right\}\right]+const={\mathbb{E}}\left[-\frac{{\left({U}_{\cdot r}\right)}^{T}\left[{\sigma }_{g}{\left({K}^{u}\right)}^{T}{K}^{u}+{\lambda }_{r}{I}_{M}\right]{U}_{\cdot r}-2{\sigma }_{g}{\left({U}_{\cdot r}\right)}^{T}{\left({K}^{u}\right)}^{T}{G}_{\cdot r}}{2}\right]+const$$

where ${I}_{M}\in {\mathbb{R}}^{M\times M}$ is the identity matrix and ${G}_{\cdot r}$ represents the $r$ column of $G$. From (20), it is found that ${U}_{\cdot r}$ follows a multivariate Gaussian distribution

21 $$q\left({U}_{\cdot r}\right)=\mathcal{N}\left({U}_{\cdot r}|{\widetilde{U}}_{\cdot r},\Sigma \left({U}_{\cdot r}\right)\right)$$

The posterior expectation ${U}_{\cdot r}$ and the covariance matrix $\Sigma \left({U}_{\cdot r}\right)$ are as follows:

$$\Sigma \left({U}_{\cdot r}\right)={\left[\widetilde{{\sigma }_{g}}{\left({K}^{u}\right)}^{T}{K}^{u}+{\widetilde{\lambda }}_{r}{I}_{M}\right]}^{-1}$$

22 $${\widetilde{U}}_{\cdot r}=\widetilde{{\sigma }_{g}}\Sigma \left({U}_{\cdot r}\right){\left({K}^{u}\right)}^{T}{\widetilde{G}}_{\cdot r}$$

Similarly, the posterior of ${V}_{\cdot r}$ follows a multivariate Gaussian distribution

23 $$q\left({V}_{\cdot r}\right)=\mathcal{N}\left({V}_{\cdot r}|{\widetilde{V}}_{\cdot r},\Sigma \left({V}_{\cdot r}\right)\right)$$

Its expectation and covariance matrix are

$$\Sigma \left({V}_{\cdot r}\right)={\left[\widetilde{{\sigma }_{h}}{\left({K}^{v}\right)}^{T}{K}^{v}+{\widetilde{\lambda }}_{r}{I}_{N}\right]}^{-1}$$

24 $${\widetilde{V}}_{\cdot r}=\widetilde{{\sigma }_{h}}\Sigma \left({V}_{\cdot r}\right){\left({K}^{v}\right)}^{T}{\widetilde{H}}_{\cdot r}$$

3) *Estimate latent variables* $G$ *and* $H$: The likelihood function in (3) contains the exponential form of ${G}_{m.}$, resulting in no conjugate prior. Therefore, referring to^35^, we utilize the following approximation.

25 $$\sigma \left(z\right)\ge \sigma \left(\xi \right)exp\left\{\frac{z-\xi }{2}-\lambda \left(\xi \right)\left({z}^{2}-{\xi }^{2}\right)\right\}, \lambda \left(\xi \right)=\frac{1}{2\xi }\left[\sigma \left(\xi \right)-\frac{1}{2}\right]$$

Then, the log likelihood of ${Y}_{m,n}$ satisfies

26 $$Ln\left[P\left({Y}_{m,n}|{G}_{m.},{H}_{n.}\right)\right]=Ln\left[{{P}_{m,n}}^{c{y}_{m,n}}{\left(1-{P}_{m,n}\right)}^{\left(1-{y}_{m,n}\right)}\right]\ge Ln\left(h({\xi }_{m,n},{G}_{m.},{H}_{n.})\right)=c{y}_{m,n}{G}_{m.}{{H}_{n.}}^{T}+(c{y}_{m,n}+1-{y}_{m,n})\left\{Ln\left[\sigma \left({\xi }_{m,n}\right)\right]-\frac{{G}_{m.}{{H}_{n.}}^{T}+{\xi }_{m,n}}{2}-\lambda \left({\xi }_{m,n}\right)\left({G}_{m.}{{H}_{n.}}^{T}{H}_{n.}{{G}_{m.}}^{T}-{{\xi }_{m,n}}^{2}\right)\right\}$$

where ${\xi }_{m,n}$ represents the local variational parameter. It can be seen that $h({\xi }_{m,n},{G}_{m.},{H}_{n.})$ is a quadratic function of ${G}_{m.}$ and is the lower bound of the log likelihood. By replacing $P\left({Y}_{m,n}|{G}_{m.},{H}_{n.}\right)$ with $h({\xi }_{m,n},{G}_{m.},{h}_{n})$ and combining (7) and (15), it can be found that the posterior of ${G}_{m.}$ satisfies the multivariate Gaussian distribution $q({G}_{m.})=\mathcal{N}({G}_{m.}|{\widetilde{G}}_{m.},\Sigma ({G}_{m.}))$, and its expectation and covariance matrix are given by (see section 2 of Appendix for details).

27 $$\begin{aligned} &{\widetilde{G}}_{m.}={\left\{\Sigma \left({\widetilde{G}}_{m.}\right)\left[{\widetilde{H}}^{T}{{a}_{m}}^{T}+\widetilde{{\sigma }_{g}}{\widetilde{U}}^{T}{\left({K}_{m\cdot }^{u}\right)}^{T}\right]\right\}}^{T}\\ &\Sigma \left({\widetilde{G}}_{m.}\right)={\left(2\sum_{n=1}^{N}\left[{b}_{m,n}{\mathbb{E}}\left({{H}_{n.}}^{T}{H}_{n.}\right)\right]+\widetilde{{\sigma }_{g}}{I}_{R}\right)}^{-1}\end{aligned}$$

where, $\widetilde{H}$ represents the expectation of $H$,${a}_{m,n}=\left(\frac{c{y}_{mn}-1+{y}_{mn}}{2}\right)$,${b}_{m,n}=\left(c{y}_{mn}+1-{y}_{mn}\right)\lambda \left({\xi }_{m,n}\right)$. Similarity, the posterior of ${H}_{n.}$ satisfies the multivariate Gaussian distribution $q\left({H}_{n.}\right)=\mathcal{N}\left({H}_{n.}|{\widetilde{H}}_{n.},\Sigma \left({H}_{n.}\right)\right)$, its expectation and covariance matrix are given by

28 $$\begin{aligned} &{\widetilde{H}}_{n.}={\left\{\Sigma \left({\widetilde{H}}_{n.}\right)\left[{\widetilde{G}}^{T}{A}_{\cdot n}+\widetilde{{\sigma }_{h}}{\widetilde{V}}^{T}{\left({K}_{n\cdot }^{v}\right)}^{T}\right]\right\}}^{T}\\ &\Sigma \left({H}_{n.}\right)={\left(2\sum_{m=1}^{M}\left[{b}_{m,n}{\mathbb{E}}\left({{G}_{m.}}^{T}{G}_{m.}\right)\right]+\widetilde{{\sigma }_{h}}{I}_{R}\right)}^{-1}\end{aligned}$$

where, $\widetilde{G}$ represents the expectation of $G$.

4) *Estimate latent variables* ${\sigma }_{g}$ *and* ${\sigma }_{h}$: Substituting (7) and (9) into (15), the approximate posterior of ${ln}q\left({\sigma }_{g}\right)$ is as follows:

29 $${ln}q\left({\sigma }_{g}\right)={\mathbb{E}}\left[ln\left\{P\left(G|U,{K}^{u},{\sigma }_{g}\right)P\left({\sigma }_{g}\right)\right\}\right]+const=\left(\frac{MR}{2}-1\right)ln\left({\sigma }_{g}\right)-{\sigma }_{g}\left(\frac{{\mathbb{E}}\left[{\Vert G-{K}^{u}U\Vert }^{2}\right]}{2}\right)+const$$

Therefore, the posterior distribution of ${\sigma }_{g}$ is a Gamma distribution with expectation

30 $${\mathbb{E}}\left[{\sigma }_{g}\right]=\frac{\widetilde{{a}^{g}}}{\widetilde{{b}^{g}}}=\frac{MR}{{\mathbb{E}}\left[{\Vert G-{K}^{u}U\Vert }^{2}\right]}$$

where, $\widetilde{{a}^{g}}$ and $\widetilde{{b}^{g}}$ are the posterior parameters of ${\sigma }_{g}$, refer to Theorem 1 in the appendix, ${\mathbb{E}}\left[{\Vert G-{K}^{u}U\Vert }^{2}\right]$ is given by

31 $${\mathbb{E}}\left[{\Vert G-{K}^{u}U\Vert }^{2}\right]={\Vert {\mathbb{E}}\left(G\right)-{K}^{u}{\mathbb{E}}\left(U\right)\Vert }^{2}+\sum_{m=1}^{M}tr\left(\Sigma \left({G}_{m\cdot }\right)\right)+tr\left({K}^{u}\sum_{r=1}^{R}\Sigma \left({U}_{\cdot r}\right){\left({K}^{u}\right)}^{T}\right)$$

Similarity, the posterior distribution of ${\sigma }_{h}$ is a Gamma distribution with expectation

32 $${\mathbb{E}}\left[{\sigma }_{h}\right]=\frac{\widetilde{{a}^{h}}}{\widetilde{{b}^{h}}}=\frac{NR}{{\mathbb{E}}\left[{\Vert H-{K}^{v}V\Vert }^{2}\right]}$$

where, $\widetilde{{a}^{h}}$ and $\widetilde{{b}^{h}}$ are the posterior parameters of ${\sigma }_{h}$, ${\mathbb{E}}\left[{\Vert H-{K}^{v}V\Vert }^{2}\right]$ is obtained similarly to formula (31).

5) *Update local variational parameter* ${\xi }_{m,n}$: According to (26), $Ln(h({\xi }_{m,n},{G}_{m.},{H}_{n.}))$ takes the derivative of ${\xi }_{m,n}$ and sets its derivative equal to 0 to obtain the optimal value of ${\xi }_{m,n}$ as follows (see section 4 of Appendix for details)

33 $${{\xi }_{m,n}}^{2}={\mathbb{E}}\left({G}_{m.}{{H}_{n.}}^{T}{H}_{n.}{{G}_{m.}}^{T}\right)={\left({\widetilde{G}}_{m.}{{\widetilde{H}}_{n.}}^{T}\right)}^{2}+vec\left(\Sigma \left({\widetilde{G}}_{m.}\right)\right){vec\left({{\widetilde{H}}_{n.}}^{T}{\widetilde{H}}_{n.}\right)}^{T}+vec\left(\Sigma \left({H}_{n.}\right)\right){vec\left({{\widetilde{G}}_{m.}}^{T}{\widetilde{G}}_{m.}\right)}^{T}+vec\left(\Sigma \left({\widetilde{G}}_{m.}\right)\right){vec\left(\Sigma \left({\widetilde{H}}_{n.}\right)\right)}^{T}$$

where, $vec\left(\cdot \right)$ represents converting a matrix into a row vector.

In summary, the optimization algorithm for solving VKBNMF is shown in Algorithm 1.

**Algorithm 1:**
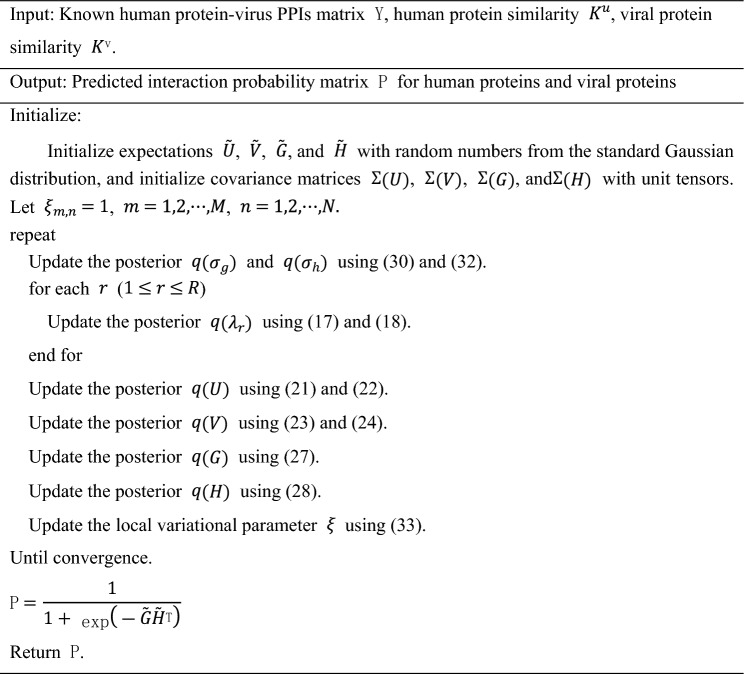
VKBNMF algorithm flow.

## Results

### Data extraction

The MorCVD database covers 19 microbial-induced cardiovascular diseases including endocarditis, myocarditis, and pericarditis, as well as 23,377 interactions between 3957 viral proteins of 432 viruses and 3202 human proteins^36^. We took vascular disease as the key word, and downloaded the human–virus PPIs of various diseases one by one from the database. To ensure that as many human (or virus) proteins as possible are covered in the dataset, we remove disease types that contain less than 100 human (or viral) proteins. Finally, the human–virus PPIs under the four disease types (corresponding to the four benchmark data sets) are obtained, as shown in Table 1.

**Table 1:** The statistics of the four datasets.

Disease name	H_num	V_num	I_num	Prop	Disease name	H_num	V_num	I_num	Prop
CI	217	410	861	0.97%	ED	557	1004	1961	0.35%
DC	424	1149	3366	0.69%	VM	898	490	4177	0.95%

“H_num” indicates the number of human proteins, “V_num” indicates the number of virus proteins, “I_num” indicates the number of interactions, “Prop” indicates the proportion of known interactions. “CI” indicates the disease “Cardiovascular Infections”, “DC” refers to “Dilated Cardiomyopathy”, “ED” refers to “Endocarditis” and “VM” refers to “Viral Myocarditis”.

From Table 1, the known interactions contained in the four benchmark datasets are very sparse (accounting for less than 1%). To obtain additional auxiliary information, we extracted amino acid sequences of these proteins from the UniProt database^37^ by R package “protr”^38^, and calculated the pseudo-amino acid composition^39^ (abbreviated as PseAAC) feature of human (or viral) proteins according to the regularization frequency of amino acids. Further, according to the PseAAC feature, KSNS is used to construct the sequence similarity of human (or viral) proteins. In summary, the four benchmark datasets in this study contain human–virus PPIs under four disease types, as well as the sequence similarity ${S}_{seq}^{h}$ (or ${S}_{seq}^{v}$) of the corresponding human (or viral) proteins.

### Experimental settings

To examine the prediction ability of the model for human–virus PPIs, new human proteins and new viral proteins, we performed fivefold crossover validation in 3 different scenarios according to previous studies^26–28,40^.

(1) “Pairwise interaction” scenario: Evaluate the predictive power with respect to human–viral PPIs. The known interactions of $Y$ are randomly divided into 5 equal parts, four of which are used for training and the remaining part is used for testing.

(2) “Human Protein” Scenario: Evaluate the predictive power with respect to human proteins. The rows of $Y$ are randomly divided into five equal parts, four of which are used for training and the remaining one is used for testing.

(3) “Viral Protein” Scenario: Evaluate the predictive power with respect to viral proteins. The columns of Y are randomly divided into five equal parts, four of which are used for training and the remaining one is used for testing.

For the “Pairwise interaction” scenario, refer to previous studies^40–43^, and select the average AUPR value, AUC value and F1 value of fivefold cross validation as evaluation indicators. For the “Human protein” and “Viral Protein” scenarios, more attention is often paid to the top-ranked candidate interactions, namely the hit rate^12,13,40^, which is calculated as follows:

34 $${\text{Hit}}\left(\uprho \right)=\frac{\left|{S}_{cand}\left(\left[\rho \cdot N\right]\right)\cap {S}_{Test}\right|}{\left|{S}_{Test}\right|}$$

where, $N$ represents the number of elements contained in the test set, $\rho$ represents the scale factor, which is {2%, 6%, 10%} in this study, and $\left[\cdot \right]$ represents rounding. ${S}_{cand}\left(\left[\rho \cdot N\right]\right)$ represents the top $\left[\rho \cdot N\right]$ PPIs with the highest predicted scores, and ${S}_{Test}$ represents the actual PPIs in the test set.

### Hyperparameter analysis

**Figure 3:**
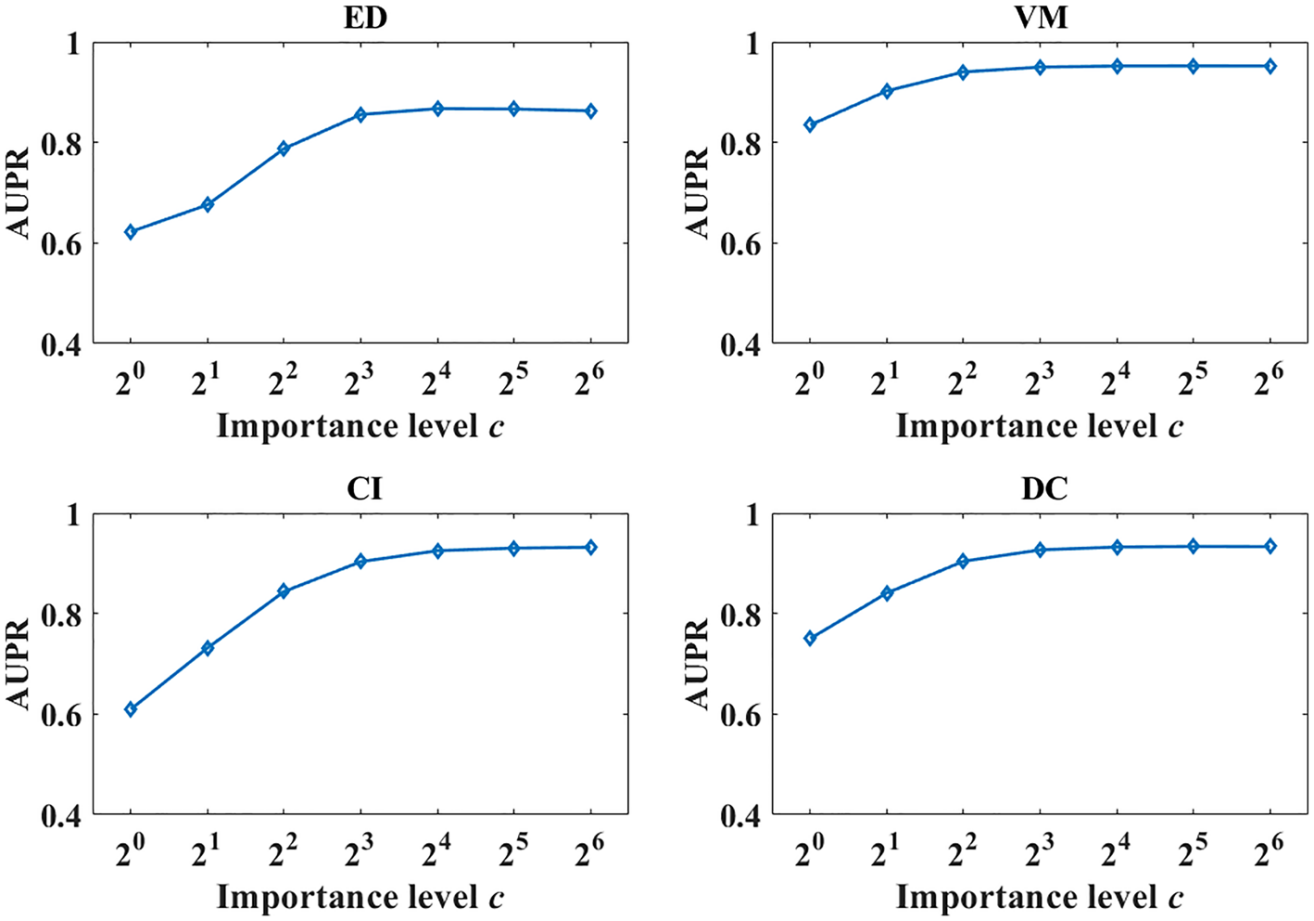
Effect of significance level c on model prediction performance.

The importance level parameter c is the only important hyperparameter of VKBNMF. To analyse the effect of c on the prediction performance, we employ the grid method. Let c be taken from $\left\{{2}^{0},{2}^{1},\ldots ,{2}^{6}\right\}$, and perform a fivefold cross validation on the four benchmark datasets for the "pair interaction" scenario, the predicted AUPR values of the model are shown in Fig. 3.

From Fig. 3, the importance level parameter $c$ has a significant effect of prediction performance on all four benchmark datasets. When $c=1$, i.e., known and unknown interactions are considered equally important, the models have the lowest AUPR on all four benchmark datasets. As $c$ increases, the prediction performance gradually improves, and when c reaches ${2}^{4}$, the AUPR of all models gradually stabilises. Therefore, this study makes the hyperparameter c take the value of 16 and performs subsequent experiments. The above analyses also show that the introduction of importance level can improve the prediction performance to some extent.

### Comparison experiments

To comprehensively evaluate the prediction performance of VKBNMF, we select 6 state-of-the-art interactive prediction models. Four advanced network models are Kernel Bayesian Matrix Factorization (KBMF)^44^, Hypergraph Logical Matrix Factorization (HGLMF)^28^, Generalized Matrix Factorization Based on Weighted Hypergraph Learning (WHGMF)^40^, Dual Laplace Regularized Least Squares (DLapRLS)^17^. Two state-of-the-art deep learning methods are Layer Attention Graph Convolutional Networks (LAGCN)^18^ and Graph Attention Networks and Dual Laplacian Regularized Least Squares (MKGAT)^45^. It should be noted that, in order to ensure the fairness of the comparison, we employ the method described in “Network construction” section to build the network for all models, and utilize the optimal parameters provided in the original code to perform prediction. Under the “Pairwise interaction” scenario, the prediction results of all models on the four benchmark datasets are shown in Table 2.

**Table 2:** Comparison of the prediction performance under “Pairwise interaction” scenario.

Dataset	Evaluation index	Methods						
		VKBNMF	KBMF	HGLMF	WHGMF	DLapRLS	LAGCN	MKGAT
CI	AUPR	**0.9101**	0.8951	0.8681	0.8685	0.8801	0.7849	0.8834
	AUC	**0.8975**	0.8785	0.8476	0.8447	0.8260	0.7201	0.8525
	F1	**0.8605**	0.8414	0.7916	0.7970	0.8299	0.7183	0.7979
DC	AUPR	**0.9316**	0.8551	0.9008	0.8639	0.9070	0.8533	0.8888
	AUC	**0.9178**	0.8344	0.8817	0.8266	0.8690	0.8365	0.8530
	F1	**0.8582**	0.7734	0.8041	0.7492	0.8321	0.7748	0.7936
ED	AUPR	**0.8727**	0.8160	0.8070	0.7703	0.8280	0.7383	0.7959
	AUC	**0.8538**	0.7930	0.7738	0.7355	0.7661	0.7032	0.7534
	F1	**0.8111**	0.7680	0.7138	0.7129	0.777	0.6822	0.7198
VM	AUPR	**0.9517**	0.9348	0.9257	0.9225	0.9337	0.8549	0.9205
	AUC	**0.9402**	0.9218	0.9078	0.8971	0.9045	0.8459	0.8938
	F1	**0.8824**	0.8566	0.8355	0.8349	0.8666	0.7858	0.8359

The numbers in bold represent the optimal values of the current indicator.

**Figure 4:**
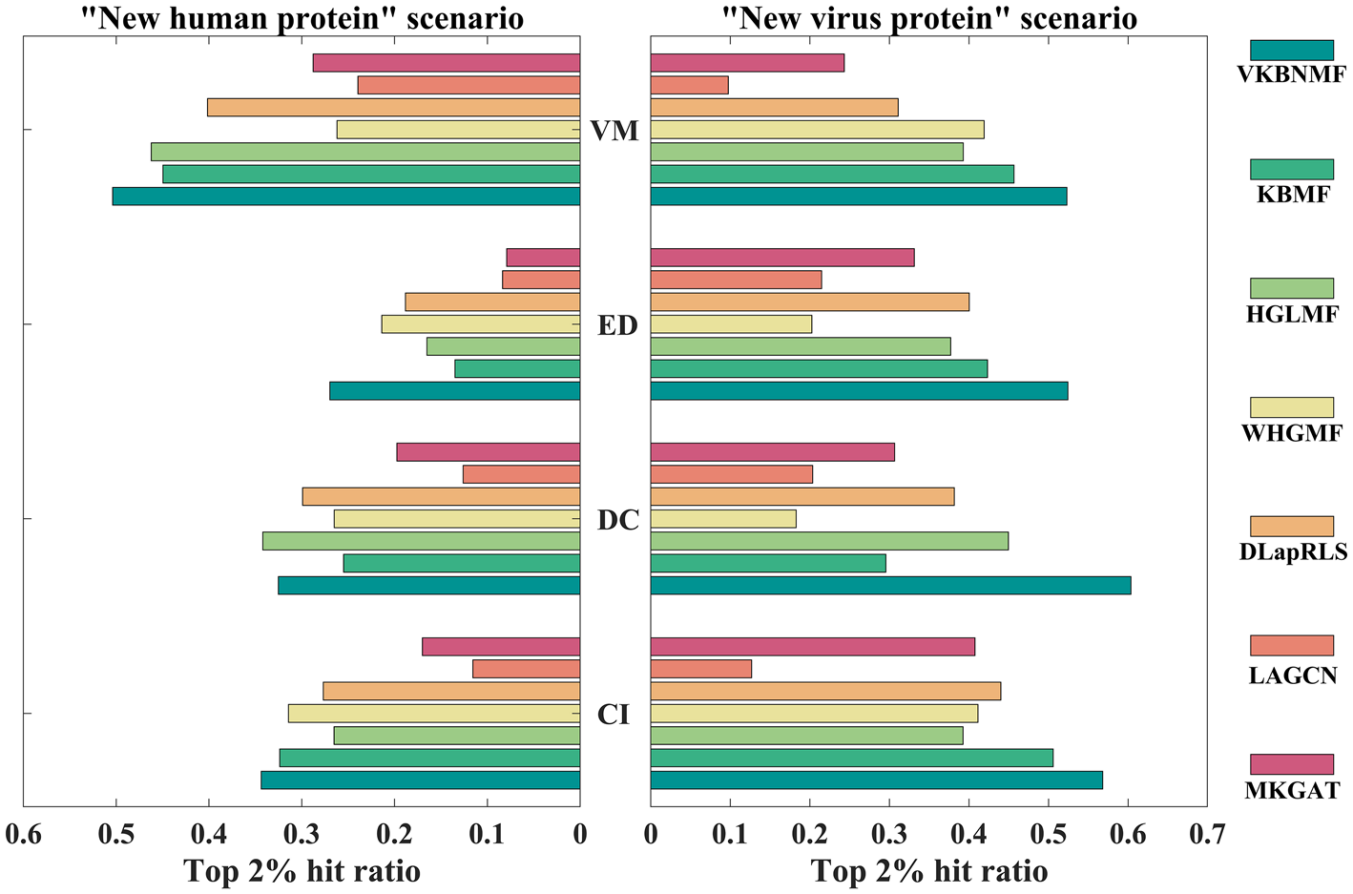
Comparison of model prediction performance for the top 2% hit rate.

From Table 2, VKBNMF shows optimal performance for all the metrics on the four benchmark datasets. On CI, VKBNMF achieves an AUPR of 0.9101, which improves 1.68%, 4.84%, 4.79%, 3.41%, 15.95%, and 3.02%, relative to KBMF's 0.8951, HGLMF's 0.8681, WHGMF's 0.8685, DLapRLS's 0.8801, LAGCN's 0.7849, and MKGAT's 0.8834, respectively. The AUPR values of VKBNMF reached 0.9316, 0.8727 and 0.9517 on CD, ED and VM, respectively, which are higher than those of other methods. Regarding the “Human protein” and “Viral Protein” scenarios, the top 2% hit rates are shown in Fig. 4.

As shown in Fig. 4, for the "new human protein" and "new virus protein" scenarios, VKBNMF shows excellent performance on most datasets. Specifically, for the "new human protein" scenario, the hit rate of VKBNMF on CI is 0.3437, which is 6.18% higher than the 0.3237 of KBMF (ranked second); the hit rate on DC is 0.3254, slightly lower than the 0.3421 of HGLM; the hit rate on ED is 0.2698, which is an increase of 26.13% compared to 0.2139 of WHGMF (ranked second); the hit rate on VM is 0.5038, which is 9% higher than 0.4622 of HGLMF (ranked second). For the “new virus protein” scenario, VKBNMF shows the best performance on all four benchmark datasets. Especially for the DC data set, the top 2% hit rate of VKBNMF exceeds 0.6. Supplemental tables S1 and S2 show the top 2%, 6%, and 10% hit rates of all methods in the "new human protein" and "new viral protein" scenarios, respectively.

In summary, whether it is “Pairwise interaction” scenario, “Human protein” scenario, or “Viral Protein” scenario, VKBNMF has shown excellent predictive performance on most data sets. The main reasons are as follows: Firstly, compared with other discriminant models, generative models (VKBNMF and KBMF) regard parameters as latent variables and realize adaptive parameter solving through variational inference, which not only avoids tedious parameter debugging, but also has considerable generalization ability and robustness. Secondly, compared with KBMF, VKBNMF improves the prediction performance by introducing nonlinear functions and importance levels into Bernoulli distributions. Finally, VKBNMF introduces automatic rank determination to realize adaptive learning of the effective dimension $R$ of the latent space, and sets an uninformative prior for the accuracy parameter to avoid manual search and improve computational efficiency.

### Robustness analysis

**Figure 5:**
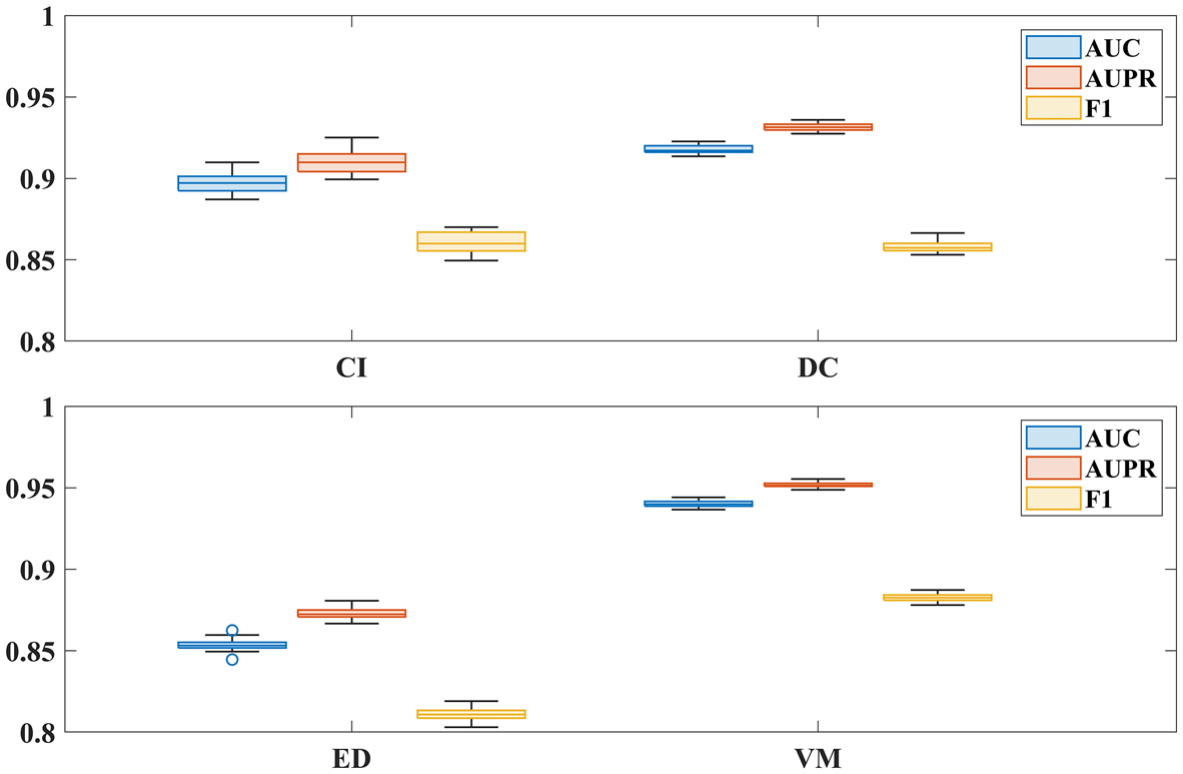
The values of AUC, AUPR, and F1 by VKBNMF under 20 random seeds of fivefold cross validation.

To assess the robustness of the models, we calculate the average AUPR, AUC and F1 values for all models under 20 different random seeds with respect to the fivefold cross-validation, and the results are shown in Table 2. We also draw a boxplot in Fig. 5, showing statistics for the AUPR, AUC, and F1 values of VKBNMF across 20 random seeds. Since the mean values on the variance of AUPR, AUC and F1 values on the four datasets are 1.644 × 10^–5^, 1.727 × 10^–5^ and 1.938 × 10^–5^, which indicates that the VKBNMF exhibits good robustness.

Furthermore, we perform paired Wilcoxon rank-sum tests of VKBNMF with other predictive models in terms of AUC, AUPR, and F1 scores, and the results are shown in Table 3. Obviously, VKBNMF significantly outperforms other prediction models at 95% confidence level (p-value < 0.05) on all datasets. It demonstrates again the significant superiority of VKBNMF in the prediction of human–viral PPIs.

**Table 3:** The P-value of the paired Wilcoxon rank sum test of VKBNMF with other predictive models.

	KBMF	HGLMF	WHGMF	DualLapRLS	LAGCN	MKGAT
CI	2.2524 × 10^–5^	1.4499 × 10^–14^	1.3356 × 10^–14^	3.0316 × 10^–13^	3.5566 × 10^–21^	4.6759 × 10^–12^
DC	6.1892 × 10^–19^	1.5543 × 10^–7^	1.3916 × 10^–14^	1.1023 × 10^–7^	5.1181 × 10^–20^	1.9812 × 10^–12^
ED	4.6422 × 10^–15^	1.4084 × 10^–18^	3.5560 × 10^–21^	2.0502 × 10^–13^	3.5560 × 10^–21^	4.3444 × 10^–21^
VM	2.7322 × 10^–7^	1.5543 × 10^–7^	1.5540 × 10^–7^	1.7414 × 10^–7^	3.5566 × 10^–21^	1.5543 × 10^–7^

## Case study

This section selects three common viruses, Epstein–Barr virus, Influenza A virus, and Human papillomavirus, as case studies to explore these viral diseases and the interaction between their viral proteins and human proteins. For each virus, we deleted all PPIs with human proteins under the corresponding disease and performed VKBNMF to obtain predicted interaction probabilities. Based on the experimental prediction scores, we obtained the top 10 PPIs with the highest probability of interacting with the virus. Then, the predicted PPIs were tested against evidence obtained from various databases of human–virus PPIs (e.g. MINT, VirHostNet, IntAct, and BioGRID, etc.). As a result, 8, 9, and 8 of the top 10 PPIs for Epstein–Barr virus, Influenza A virus, and Human papillomavirus were verified, respectively.

Epstein–Barr virus (EBV), also known as Human gammaherpesvirus 4, is a member of the herpesvirus family, which is a double-stranded DNA virus and one of the most common human viruses^46^. EBV is found all over the world, which is generally transmitted through body fluids, mainly saliva. This virus is closely related to non-gonorrheal malignancy such as gastric cancer and nasopharyngeal cancer^47^, as well as children's Alice in Wonderland syndrome^48^ and acute cerebellar ataxia^49^. Calderwood et al.^50^ found that human proteins targeted by EBV proteins are rich in highly connected or hub proteins, and the targeting center may be an effective mechanism for EBV recombination in cellular processes. In this study, all interactions between Epstein–Barr virus (Taxonomy ID is 10376) and human proteins under Cardiovascular Infections were deleted, and 8 of the top 10 PPIs predicted by VKBNMF were verified, as shown in Table 4.

**Table 4:** The top 10 PPIs of Epstein–Barr virus identified by VKBNMF.

Virus protein	Human protein	Score	Dataset
Q3KSU8	P28799	0.9302	IntAct
P0C732	P12004	0.9267	Unconfirmed
P03186	P28799	0.9002	VirHostNet
P0C732	P50402	0.8871	Unconfirmed
P0C732	O95817	0.8468	BioGRID
P0C732	O95071	0.8371	BioGRID
P0C732	P04792	0.7912	BioGRID
G3CKS7	O95071	0.7849	VirHostNet
P0C736	P02751	0.7272	IntAct
P0C762	P04275	0.7261	IntAct

Influenza A subtype H5N1 is a subtype of influenza A virus that causes disease in humans and many other species^51^. Handling infected poultry is a risk factor for H5N1 infection, and about 60 percent of humans known to be infected with the Asian strain of H5N1 have died from the virus. Furthermore, H5N1 may mutate or recombine into a strain capable of efficient human-to-human transmission^52^. Due to its high lethality, endemic existence, and continuous major mutations, H5N1 was once considered the world's greatest pandemic threat, and countries around the world spent a lot of manpower and material resources on H5N1 research. In this study, all interactions between H5N1 (Taxonomy ID is 284218) and human proteins were deleted under Viral Myocarditis disease, and 9 of the top 10 PPIs with interaction probability predicted by VKBNMF were verified, as shown in Table 5.

**Table 5:** The top 10 PPIs of Influenza A virus identified by VKBNMF.

Virus protein	Human protein	Score	Dataset
Q5EP28	Q8WV44	0.9663	IntAct
Q5EP28	O95232	0.9521	IntAct
Q5EP28	Q8TAE8	0.9520	Unconfirmed
Q5EP28	Q4G0J3	0.9502	IntAct
Q5EP28	Q96EY7	0.9014	IntAct
Q5EP28	Q9BYD6	0.8913	IntAct
Q5EP28	Q9NYK5	0.8872	IntAct
Q5EP28	O76021	0.8818	IntAct
Q5EP28	Q9P015	0.8756	IntAct
Q5EP28	Q9Y3B7	0.8729	IntAct

Human papillomavirus (HPV) infection is one of the most common sexually transmitted diseases and has been associated with cancers such as cervical, head and neck squamous cell carcinoma (HNSCC), and anal cancer^53^. HPV infection is mainly transmitted through skin-to-skin or skin-to-mucosal contact^54^. HPV 16 is the most common high-risk type of HPV, which causes a trusted source of 50% of cervical cancers worldwide, and usually does not cause any noticeable symptoms, although it can bring about cervical changes^55^. In this study, all interactions between HPV 16 (Taxonomy ID is 333760) and human proteins were deleted under Viral Myocarditis disease, and 8 of the top 10 PPIs with interaction probability predicted by VKBNMF were verified, as shown in Table 6.

**Table 6:** The top 10 PPIs of Influenza A virus identified by VKBNMF.

Virus protein	Human protein	Score	Dataset
P03120	P11021	0.9663	MINT
P03129	Q9P0J7	0.9545	MINT
P03129	P47869	0.9403	VirHostNet
P03120	P20226	0.9350	MINT
P03129	O00203	0.9348	Unconfirmed
P03129	Q14671	0.9133	VirHostNet
P03129	Q15678	0.8783	VirHostNet
P03129	Q96C00	0.8648	VirHostNet
P03120	P04637	0.8485	MINT
P03129	Q9NP81	0.8471	Unconfirmed

## Discussion

This study proposes a novel human–virus PPIs prediction method named kernel Bayesian nonlinear matrix factorization based on variational inference (VKBNMF). The novelty of this method is to establish a Bayesian framework of nonlinear matrix factorization and introduce auxiliary information to improve the predictive ability of new proteins. Meanwhile, VKBNMF takes model parameters as latent variables, and realizes the adaptive solution of parameters by inferring its posterior probability, avoiding tedious parameter debugging and enhancing the generalization ability of the model. In addition, this study builds a variational framework for model solving, which ensures the efficiency of solving large-scale data.

To evaluate the performance of VKBNMF, we conducted extensive experiments on multiple benchmark datasets and various experimental scenarios. The experimental results found that for the “Pairwise interaction” scenario, except for the CI dataset, VKBNMF achieved better AUPR, AUC and F1 values on the other three datasets. Under the “Human protein” scenario, the hit rates of VKBNMF are slightly lower than those of KBMF and HGLMF on CI and DC datasets, respectively, and VKBNMF achieves significantly higher hit rates on the remaining two datasets. Under the “Viral Protein” scenario, VKBNMF showed a higher hit rate on all four benchmarks. Finally, we take three common viruses as case studies to further verify the effectiveness of our method.

However, VKBNMF still has some aspects worthy of further study. Firstly, to facilitate the solution of the model, we select common conjugate priors, such as multivariate Gaussian distribution and Gamma distribution. The following research plans to try some other effective prior distributions. Secondly, for the purpose of model evaluation, we separately studied human–virus PPIs in different diseases, ignoring the relationship between different diseases. In the future, we plan to establish an integrated prediction model combining disease types and human–virus PPIs.

### Supplementary Information

Supplementary Information 1.

## Data Availability

VKBNMF is freely available in a GitHub repository (https://github.com/Mayingjun20179/VKBNMF).
