# Supplementary material for: Kernel Bayesian nonlinear matrix factorization based on variational inference for human–virus protein–protein interaction prediction
Source: Sci Rep. 2024 Mar 8;14:5693. doi: 10.1038/s41598-024-56208-w (PMC10920681; doi:10.1038/s41598-024-56208-w)
Supplement: Supplementary file 1 — Supplementary Information 1. [file 41598_2024_56208_MOESM1_ESM.docx]

# 1 The variational posterior distribution of $\boldsymbol{U}$and $\boldsymbol{V}$

| $Lnq\left( U_{\cdot r} \right)=\mathbb{E}_{q\left( \Theta\backslash U_{\cdot r} \right)}\left[ Ln\left\{ P\left( G \vert U,K^{u},\sigma_{g}^{2} \right)P\left( U \vert\lambda\right) \right\} \right]+const=\mathbb{E}\left[ \sum_{m=1}^{M} \sum_{r=1}^{R} \left( -\frac{\sigma_{g}\left[ \left( K_{m\cdot}^{u}U_{\cdot r} \right)^{T}-G_{m,r} \right]\left[ K_{m\cdot}^{u}U_{\cdot r}-G_{m,r} \right]}{2}-\frac{\lambda_{r}\left( U_{m,r} \right)^{2}}{2} \right) \right]+const=\mathbb{E}\left[ \sum_{m=1}^{M} \left( -\frac{\sigma_{g}\left[ \left( K_{m\cdot}^{u}U_{\cdot r} \right)^{T}-G_{m,r} \right]\left[ K_{m\cdot}^{u}U_{\cdot r}-G_{m,r} \right]}{2}-\frac{\lambda_{r}\left( U_{m,r} \right)^{2}}{2} \right) \right]+const=\mathbb{E}\left[ \sum_{m=1}^{M} \left( -\frac{\sigma_{g}\left( U_{\cdot r} \right)^{T}\left( K_{m\cdot}^{u} \right)^{T}K_{m\cdot}^{u}U_{\cdot r}-2\sigma_{g}\left( U_{\cdot r} \right)^{T}\left( K_{m\cdot}^{u} \right)^{T}G_{m,r}}{2}-\frac{\lambda_{r}\left( U_{m,r} \right)^{2}}{2} \right) \right]+const=\mathbb{E}\left[ -\frac{\sigma_{g}\left( U_{\cdot r} \right)^{T}\left[ \left( K^{u} \right)^{T}K^{u} \right]U_{\cdot r}-2\sigma_{g}\left( U_{\cdot r} \right)^{T}\left( K^{u} \right)^{T}G_{\cdot r}}{2}-\frac{\lambda_{r}\left( U_{\cdot r} \right)^{T}U_{\cdot r}}{2} \right]+const=\mathbb{E}\left[ -\frac{\left( U_{\cdot r} \right)^{T}\left[ \sigma_{g}\left( K^{u} \right)^{T}K^{u}+\lambda_{r}I_{M} \right]U_{\cdot r}-2\sigma_{g}\left( U_{\cdot r} \right)^{T}\left( K^{u} \right)^{T}G_{\cdot r}}{2} \right]+const$ | (1) |
| --- | --- |

Hence, we observe that $q\left( U_{\cdot r} \right)$ is also a multivariate Gaussian distribution and the posterior parameters can be easily obtained from the above Gaussian form.

# 2 The variational posterior distribution of $\boldsymbol{G}$ and $\boldsymbol{H}$

The log-likelihood function of $Y_{m,n}$ is

| $Ln\left[ P\left( Y_{m,n} \vert G_{m.},H_{n.} \right) \right]=Ln\left[ {P_{m,n}}^{cy_{m,n}}\left( 1-P_{m,n} \right)^{\left( 1-y_{m,n} \right)} \right]=cy_{m,n}G_{m.}{H_{n.}}^{T}+(cy_{m,n}+1-y_{m,n})Ln\left[ \sigma\left( -G_{m.}{H_{n.}}^{T} \right) \right]$ | (2) |
| --- | --- |

According to the following approximation

| $\sigma\left( z \right)\geq\sigma\left( \xi\right)exp\left\{ \frac{z-\xi}{2}-\lambda\left( \xi\right)\left( z^{2}-\xi^{2} \right) \right\}$  $\lambda\left( \xi\right)=\frac{1}{2\xi}\left[ \sigma\left( \xi\right)-\frac{1}{2} \right]$ | (3) |
| --- | --- |

Then, the log likelihood of $Y_{m,n}$ satisfies

| $Ln\left[ P\left( Y_{m,n} \vert G_{m.},H_{n.} \right) \right]\geq Ln\left( h\left（ \xi_{m,n},G_{m.},H_{n.} \right） \right)=cY_{m,n}G_{m.}{H_{n.}}^{T}+(cY_{m,n}+1-Y_{m,n})\left\{ Ln\left[ \sigma\left( \xi_{m,n} \right) \right]-\frac{G_{m.}{H_{n.}}^{T}+\xi_{m,n}}{2}-\lambda\left( \xi_{m,n} \right)\left( G_{m.}{H_{n.}}^{T}H_{n.}{G_{m.}}^{T}-{\xi_{m,n}}^{2} \right) \right\}=\left( \frac{cY_{m,n}-1+Y_{m,n}}{2} \right)G_{m.}{H_{n.}}^{T}-(cY_{m,n}+1-Y_{m,n})\lambda\left( \xi_{m,n} \right)G_{m.}{H_{n.}}^{T}H_{n.}{G_{m.}}^{T}+\left( cY_{m,n}+1-Y_{m,n} \right)Ln\left[ \sigma\left( \xi_{m,n} \right) \right]-(cY_{m,n}+1-Y_{m,n})\frac{\xi_{m,n}}{2}+(cY_{m,n}+1-Y_{m,n})\lambda\left( \xi_{m,n} \right){\xi_{m,n}}^{2}$ | (4) |
| --- | --- |

Replacing $P\left( Y_{m,n} | G_{m.},H_{n.} \right)$ with $h\left（ \xi_{m,n},G_{m.},H_{n.} \right）$ and combining $P\left( G | U,K^{u},\sigma_{g} \right)$, we get

| $\mathbb{E}_{q\left( \Theta\backslash G_{m.} \right)}\left[ Ln\left\{ h\left（ \xi_{m,n},G_{m.},H_{n.} \right）P\left( G \vert U,K^{u},\sigma_{g}^{2} \right) \right\} \right]=\mathbb{E}_{q\left( \Theta\backslash G_{m.} \right)}.\left\{ \sum_{n=1}^{N} \left[ \left( \frac{cy_{mn}-1+y_{mn}}{2} \right)G_{m.}{H_{n.}}^{T}-\left( cy_{mn}+1-Y_{m,n} \right)\lambda\left( \xi_{m,n} \right)G_{m.}{H_{n.}}^{T}H_{n.}{G_{m.}}^{T} \right]-\frac{\sigma_{g}{\left( G_{m.}-K_{m\cdot}^{u}U \right)\left( G_{m.}-K_{m\cdot}^{u}U \right)}^{T}}{2} \right\}$ | (5) |
| --- | --- |

For simplicity, let $A_{m,n}=\left( \frac{cy_{mn}-1+y_{mn}}{2} \right)$, $B_{m,n}=\left( cy_{mn}+1-Y_{m,n} \right)\lambda\left( \xi_{m,n} \right)$, (5) be converted to

| $\mathbb{E}_{q\left( \Theta\backslash G_{m.} \right)}\left[ Ln\left\{ h\left（ \xi_{m,n},G_{m.},H_{n.} \right）P\left( G \vert U,K^{u},\sigma_{g}^{2} \right) \right\} \right]=\mathbb{E}_{q\left( \Theta\backslash G_{m.} \right)}.\left\{ \sum_{n=1}^{N} \left[ A_{m,n}G_{m.}{H_{n.}}^{T}-B_{m,n}G_{m.}{H_{n.}}^{T}H_{n.}{G_{m.}}^{T} \right]-\frac{\sigma_{g}{\left( G_{m.}-K_{m\cdot}^{u}U \right)\left( G_{m.}-K_{m\cdot}^{u}U \right)}^{T}}{2} \right\}=G_{m.}\left\{ \tilde{H}^{T}{A_{m\cdot}}^{T}+\tilde{\sigma_{g}}U^{T}\left( K_{m\cdot}^{u} \right)^{T} \right\}-G_{m.}\left\{ \frac{2\sum_{n=1}^{N} \left[ B_{m,n}\mathbb{E}\left( {H_{n.}}^{T}H_{n.} \right) \right]+\tilde{\sigma_{g}}I_{R}}{2} \right\}{G_{m.}}^{T}$ | (6) |
| --- | --- |

Hence, we observe that $q\left( G_{m.} \right)$ is also a multivariate Gaussian distribution and the posterior parameters can be easily obtained from the above Gaussian form.

# 3 Prove theorem 1

| $\mathbb{E}\left[ \left\Vert G-K^{u}U \right\Vert^{2} \right]\mathbb{=E}\left[ tr\left[ \left( G-K^{u}U \right)\left( G^{T}-U^{T}\left( K^{u} \right)^{T} \right) \right] \right]\mathbb{=E}\left[ tr\left( GG^{T}+K^{u}UU^{T}\left( K^{u} \right)^{T}-2K^{u}UG^{T} \right) \right]=tr\left( \mathbb{E}\left[ GG^{T} \right]+K^{u}\mathbb{E}\left( UU^{T} \right)\left( K^{u} \right)^{T}-2K^{u}\mathbb{E}\left( U \right){\mathbb{E}\left( G \right)}^{T} \right)=tr\left( \mathbb{E}\left( G \right)\mathbb{E}\left( G \right)^{T}+diag\left[ \begin{matrix} tr\left( \Sigma\left( G_{1\cdot} \right) \right) & \cdots& tr\left( \Sigma\left( G_{M\cdot} \right) \right) \end{matrix} \right]+K^{u}\left\{ \mathbb{E}\left( U \right){\mathbb{E}\left( U \right)}^{T}+\sum_{r=1}^{R} \Sigma\left( U_{\cdot r} \right) \right\}\left( K^{u} \right)^{T} \right)=tr\left\{ \mathbb{E}\left( G \right)\mathbb{E}\left( G \right)^{T}+K^{u}\mathbb{E}\left( U \right){\mathbb{E}\left( U \right)}^{T}\left( K^{u} \right)^{T}-2K^{u}\mathbb{E}\left( U \right){\mathbb{E}\left( G \right)}^{T} \right\}+tr\left( diag\left[ \begin{matrix} tr\left( \Sigma\left( G_{1\cdot} \right) \right) & \cdots& tr\left( \Sigma\left( G_{M\cdot} \right) \right) \end{matrix} \right] \right)+tr\left( K^{u}\sum_{r=1}^{R} \Sigma\left( U_{\cdot r} \right)\left( K^{u} \right)^{T} \right)=\left\Vert\mathbb{E}\left( G \right)-K^{u}\mathbb{E}\left( U \right) \right\Vert^{2}+\sum_{m=1}^{M} tr\left( \Sigma\left( G_{m\cdot} \right) \right)+tr\left( K^{u}\sum_{r=1}^{R} \Sigma\left( U_{\cdot r} \right)\left( K^{u} \right)^{T} \right)$ | (7) |
| --- | --- |

# 4 Solution of local variational parameter $\boldsymbol{\xi}_{\mathbf{m,n}}$

Let $L\left( \xi_{m,n} \right)$ denote $\mathbb{E}\left\{ Ln\left( h\left（ \xi_{m,n},G_{m.},H_{n.} \right） \right) \right\}$, get

| $L\left( \xi_{m,n} \right)\mathbb{=E}\left\{ Ln\left( h\left（ \xi_{m,n},G_{m.},H_{n.} \right） \right) \right\}\mathbb{=E}\left\{ \left( cY_{m,n}+1-Y_{m,n} \right)\left\{ Ln\left[ \sigma\left( \xi_{m,n} \right) \right]-\frac{G_{m.}{H_{n.}}^{T}+\xi_{m,n}}{2}-\lambda\left( \xi_{m,n} \right)\left( G_{m.}{H_{n.}}^{T}H_{n.}{G_{m.}}^{T}-{\xi_{m,n}}^{2} \right) \right\} \right\}+const$ | (8) |
| --- | --- |

It can be seen that $\left( cY_{m,n}+1-Y_{m,n} \right)\geq1$ has no effect on the value of $\xi_{m,n}$. Simplify $L\left( \xi_{m,n} \right)$ to

| $\tilde{L}\left( \xi_{m,n} \right)=Ln\left[ \sigma\left( \xi_{m,n} \right) \right]-\frac{\xi_{m,n}}{2}-\lambda\left( \xi_{m,n} \right)\left( \mathbb{E}\left[ G_{m.}{H_{n.}}^{T}H_{n.}{G_{m.}}^{T} \right]-{\xi_{m,n}}^{2} \right)+const$ | (9) |
| --- | --- |

Take the partial derivative of $\tilde{L}\left( \xi_{m,n} \right)$ with respect to $\xi_{m,n}$, and get

| $\frac{d\tilde{L}\left( \xi_{m,n} \right)}{d\xi_{m,n}}=\frac{\sigma^{'}\left( \xi_{m,n} \right)}{\sigma\left( \xi_{m,n} \right)}-\frac{1}{2}\mathbb{-E}\left[ G_{m.}{H_{n.}}^{T}H_{n.}{G_{m.}}^{T} \right]\lambda^{'}\left( \xi_{m,n} \right)+\lambda^{'}\left( \xi_{m,n} \right){\xi_{m,n}}^{2}+2\xi_{m,n}\lambda\left( \xi_{m,n} \right)$ | (10) |
| --- | --- |

According to the definition of $\sigma\left( \cdot\right)$ and $\lambda\left( \cdot\right)$, then

| $\sigma^{'}\left( \xi_{m,n} \right)=\sigma\left( \xi_{m,n} \right)\left( 1-\sigma\left( \xi_{m,n} \right) \right)$  $2\xi_{m,n}\lambda\left( \xi_{m,n} \right)=\sigma\left( \xi_{m,n} \right)-\frac{1}{2}$ | (11) |
| --- | --- |

Letting the derivative in (10) equal to 0, which transforms into

| $\frac{\sigma^{'}\left( \xi_{m,n} \right)}{\sigma\left( \xi_{m,n} \right)}-\frac{1}{2}\mathbb{-E}\left[ G_{m.}{H_{n.}}^{T}H_{n.}{G_{m.}}^{T} \right]\lambda^{'}\left( \xi_{m,n} \right)+\lambda^{'}\left( \xi_{m,n} \right){\xi_{m,n}}^{2}+2\xi_{m,n}\lambda\left( \xi_{m,n} \right)=0$  $\Longleftrightarrow1-\sigma\left( \xi_{m,n} \right)-\frac{1}{2}-\lambda^{'}\left( \xi_{m,n} \right)\mathbb{E}\left[ G_{m.}{H_{n.}}^{T}H_{n.}{G_{m.}}^{T} \right]+\lambda^{'}\left( \xi_{m,n} \right){\xi_{m,n}}^{2}+\sigma\left( \xi_{m,n} \right)-\frac{1}{2}=0$  $\Longleftrightarrow\lambda^{'}\left( \xi_{m,n} \right)\left( {\xi_{m,n}}^{2}\mathbb{-E}\left[ G_{m.}{H_{n.}}^{T}H_{n.}{G_{m.}}^{T} \right] \right)=0$ | (12) |
| --- | --- |

Reference [1], $\lambda^{'}\left( \xi_{m,n} \right)\neq0$, then

| ${\xi_{m,n}}^{2}\mathbb{=E}\left( G_{m.}{H_{n.}}^{T}H_{n.}{G_{m.}}^{T} \right)==\left( \tilde{G}_{m.}{\tilde{H}_{n.}}^{T} \right)^{2}+vec\left( \Sigma\left( \tilde{G}_{m.} \right) \right){vec\left( {\tilde{H}_{n.}}^{T}\tilde{H}_{n.} \right)}^{T}+vec\left( \Sigma\left( H_{n.} \right) \right){vec\left( {\tilde{G}_{m.}}^{T}\tilde{G}_{m.} \right)}^{T}+vec\left( \Sigma\left( \tilde{G}_{m.} \right) \right){vec\left( \Sigma\left( \tilde{H}_{n.} \right) \right)}^{T}$ | (13) |
| --- | --- |

Supplementary Table S1. Comparison of the prediction performance under “Human protein” scenario

| Dataset | Hit rate | Methods | | | | | | |
| --- | --- | --- | --- | --- | --- | --- | --- | --- |
|  |  | KBLMF | KBMF | HGLMF | CIBM | DLapRLS | LAGCN | MKGAT |
| CI | 2% | 0.3208 | **0.3237** | 0.2653 | 0.3143 | 0.2769 | 0.1156 | 0.1702 |
|  | 6% | 0.4234 | **0.5042** | 0.3885 | 0.3970 | 0.3393 | 0.2034 | 0.2512 |
|  | 10% | 0.4744 | **0.5713** | 0.4398 | 0.4538 | 0.3710 | 0.2783 | 0.2781 |
| DC | 2% | 0.3246 | 0.2550 | **0.3421** | 0.2651 | 0.2992 | 0.1261 | 0.1976 |
|  | 6% | **0.4660** | 0.3784 | 0.4477 | 0.3704 | 0.3616 | 0.2173 | 0.2777 |
|  | 10% | **0.5263** | 0.4507 | 0.5072 | 0.4331 | 0.3859 | 0.2780 | 0.3287 |
| ED | 2% | **0.2509** | 0.1349 | 0.1651 | 0.2139 | 0.1884 | 0.0838 | 0.0791 |
|  | 6% | **0.3432** | 0.2882 | 0.2454 | 0.2928 | 0.2392 | 0.1389 | 0.1486 |
|  | 10% | **0.4060** | 0.3865 | 0.2915 | 0.3522 | 0.2652 | 0.1931 | 0.1961 |
| VM | 2% | **0.5104** | 0.4496 | 0.4622 | 0.2621 | 0.4016 | 0.2396 | 0.2877 |
|  | 6% | **0.6610** | 0.5943 | 0.5796 | 0.3830 | 0.4742 | 0.3586 | 0.3948 |
|  | 10% | **0.7079** | 0.6546 | 0.6420 | 0.4467 | 0.5056 | 0.4629 | 0.4527 |

Supplementary Table S2. Comparison of the prediction performance under “Virus protein” scenario

| Dataset | Hit rate | Methods | | | | | | |
| --- | --- | --- | --- | --- | --- | --- | --- | --- |
|  |  | KBLMF | KBMF | HGLMF | CIBM | DLapRLS | LAGCN | MKGAT |
| CI | 2% | **0.5486** | 0.5058 | 0.3924 | 0.4112 | 0.44 | 0.1267 | 0.4075 |
|  | 6% | **0.6696** | 0.6367 | 0.5171 | 0.5294 | 0.4858 | 0.2718 | 0.4907 |
|  | 10% | **0.7102** | 0.6798 | 0.5883 | 0.6013 | 0.5136 | 0.3617 | 0.5217 |
| DC | 2% | **0.5900** | 0.2956 | 0.4495 | 0.1827 | 0.3814 | 0.2035 | 0.3064 |
|  | 6% | **0.7146** | 0.4725 | 0.5611 | 0.2921 | 0.4439 | 0.3532 | 0.3966 |
|  | 10% | **0.7529** | 0.5717 | 0.6096 | 0.4069 | 0.4725 | 0.4463 | 0.4525 |
| ED | 2% | **0.5284** | 0.4233 | 0.377 | 0.2024 | 0.4002 | 0.2146 | 0.3312 |
|  | 6% | **0.6229** | 0.5343 | 0.4609 | 0.279 | 0.4457 | 0.3694 | 0.393 |
|  | 10% | **0.6549** | 0.5876 | 0.5019 | 0.3854 | 0.4645 | 0.4603 | 0.4326 |
| VM | 2% | **0.5146** | 0.4565 | 0.3928 | 0.4189 | 0.311 | 0.0975 | 0.2432 |
|  | 6% | **0.6606** | 0.5965 | 0.523 | 0.5312 | 0.382 | 0.2522 | 0.3386 |
|  | 10% | **0.7106** | 0.6542 | 0.5785 | 0.5932 | 0.413 | 0.3539 | 0.3853 |

# Reference

[1] T. S. JAAKKOLA, and M. I. JORDAN, “Bayesian parameter estimation via variational methods,” *Statistics and Computing,* vol. 10, no. 25-37, 2000.
